# Supplementary material for: Amino Acid Profiling of Follicular Fluid in Assisted Reproduction Reveals Important Roles of Several Amino Acids in Patients with Insulin Resistance
Source: Int J Mol Sci. 2023 Aug 5;24(15):12458. doi: 10.3390/ijms241512458 (PMC10419978; doi:10.3390/ijms241512458)
Supplement: Supplementary file 1 [file ijms-24-12458-s001.zip › ijms-2514224-supplementary.pdf]

	Concentration of amino acids (µmol/L)																			
**Sample ID**	ASP	GLU	ASN	SER	GLN	HIS	GLY	THR	ASN	ALA	TYR	CYS	VAL	MET	TRP	PHE	ILE	LEU	LYS	PRO
**IR1**	4.9	39.4	32.6	44.9	382.0	50.3	104.3	117.7	36.5	241.5	31.4	26.6	160.8	24.4	61.9	38.0	45.6	78.0	110.8	265.4
**IR2**	6.5	6.6	46.8	54.6	449.4	65.4	148.0	126.2	29.9	334.3	50.9	22.3	153.4	30.4	57.7	47.4	47.1	75.5	142.3	394.2
**IR3**	5.4	67.9	22.7	24.1	283.9	53.2	94.9	79.0	15.5	224.5	23.4	16.5	137.4	18.2	57.2	33.4	27.1	50.0	83.1	204.7
**IR4**	13.7	62.3	43.8	68.7	421.1	56.0	218.0	126.6	30.0	279.5	24.6	22.7	139.4	19.7	39.2	36.3	29.9	58.6	92.3	132.0
**IR5**	7.5	103.3	33.7	45.4	352.3	61.5	100.3	78.7	36.5	339.3	27.9	25.1	170.9	18.1	49.6	42.7	30.9	61.1	100.4	119.7
**IR6**	6.9	64.5	29.9	59.7	352.9	46.9	134.1	140.1	46.7	274.0	50.3	32.0	187.5	19.1	46.1	42.9	42.5	73.5	115.9	186.0
**IR7**	8.0	123.9	55.5	100.9	405.2	78.0	137.5	170.1	35.7	276.8	26.6	74.0	246.9	75.5	71.9	58.1	52.1	89.3	139.9	178.0
**IR8**	13.1	77.5	45.4	60.1	427.7	80.8	96.7	167.3	33.9	271.3	68.3	11.3	298.5	31.6	52.9	61.4	71.6	133.6	129.2	430.7
**IR9**	10.7	60.2	26.0	35.8	236.0	50.5	52.0	84.4	19.3	178.8	22.1	15.4	123.2	14.1	39.0	39.9	26.7	47.6	69.6	98.7
**IR10**	10.7	93.3	30.2	63.5	344.3	42.3	148.5	133.1	32.3	336.7	50.2	17.4	224.7	21.2	47.2	50.4	41.2	79.6	111.9	224.8
**IR11**	11.5	69.9	41.7	49.3	495.3	66.0	158.7	131.6	49.2	371.6	42.8	21.1	179.8	23.5	54.8	54.5	37.8	66.0	102.2	202.3
**NIR1**	5.8	63.3	32.7	55.7	434.4	60.8	149.3	131.0	34.0	244.1	31.1	38.8	135.8	19.5	54.4	40.7	38.6	60.9	90.3	173.9
**NIR2**	4.7	57.8	32.4	66.5	408.1	56.1	176.0	107.7	36.3	236.9	29.2	34.6	131.7	16.7	51.9	37.3	28.9	51.0	87.6	138.8
**NIR3**	7.4	81.0	35.0	64.2	467.0	73.5	221.5	161.8	32.0	310.0	30.4	25.3	224.1	19.1	65.8	43.8	61.8	95.3	116.9	128.3
**NIR4**	10.0	90.7	32.6	53.6	394.4	63.8	170.0	126.0	34.4	225.6	30.3	32.9	164.5	15.6	48.9	46.4	35.6	74.5	119.5	105.2
**NIR5**	10.6	80.8	42.9	69.3	589.2	66.4	245.8	149.8	53.8	246.5	30.5	27.4	102.5	18.2	47.1	36.3	25.7	45.6	75.1	141.5
**NIR6**	7.1	56.7	49.6	81.5	527.3	70.1	181.8	196.7	39.6	276.8	24.8	34.8	160.4	21.2	50.7	40.3	37.5	62.2	116.0	158.3
**NIR7**	6.8	75.8	48.8	83.0	414.1	65.4	262.3	151.0	39.4	223.3	27.1	27.3	145.4	17.5	35.0	38.0	39.3	69.7	126.8	132.7
**NIR8**	7.6	64.5	42.0	65.6	516.3	65.7	211.8	158.3	44.3	275.1	33.0	36.7	127.5	22.6	49.9	47.4	29.6	55.0	118.5	228.5

**NIR9**	4.7	49.0	54.9	96.3	768.8	80.8	333.0	191.7	76.4	273.2	35.7	40.5	170.5	25.7	42.0	50.8	46.0	97.4	155.6	171.1
**NIR10**	5.2	50.1	38.0	55.2	375.2	60.9	139.9	129.3	28.2	203.7	25.4	26.2	140.0	18.5	53.6	40.3	42.0	68.0	84.0	100.8
**NIR11**	6.2	60.2	25.5	40.8	310.4	52.9	97.8	95.8	18.5	172.2	19.7	19.1	128.6	14.8	55.1	35.0	30.7	46.9	62.5	107.2
**NIR12**	5.0	43.7	39.4	62.4	388.3	76.8	140.8	69.0	40.9	188.3	33.3	63.7	141.3	30.0	57.1	55.7	35.9	67.6	108.6	197.2
**NIR13**	3.0	16.6	43.3	78.8	413.6	76.6	163.2	126.8	54.2	272.5	55.1	24.9	227.0	29.3	63.9	60.3	51.4	104.6	170.1	285.1
**NIR14**	4.3	35.3	38.0	66.8	354.0	65.1	177.0	103.0	38.2	192.7	31.0	15.5	226.1	18.3	65.2	48.4	46.2	81.7	93.9	128.7
**NIR15**	10.1	93.8	39.9	60.8	509.3	67.2	254.6	128.7	30.3	288.5	26.3	30.7	135.7	21.8	59.1	39.2	31.7	48.7	84.3	290.9
**NIR16**	6.7	67.3	39.4	75.6	371.5	58.5	177.1	112.8	55.7	258.0	31.7	20.4	169.3	20.6	48.0	39.9	39.5	70.9	113.6	128.3
**NIR17**	5.4	42.4	46.8	68.4	439.8	75.4	179.3	213.8	41.5	266.4	36.9	31.1	182.9	26.7	60.9	49.7	45.4	79.7	124.5	125.7
**NIR18**	4.7	53.3	30.5	53.6	462.3	64.3	148.9	106.2	50.1	264.7	30.7	24.1	164.6	24.1	60.9	34.6	34.2	67.1	129.8	269.3
**NIR19**	8.2	80.4	32.8	46.8	329.7	66.8	140.5	86.9	33.4	309.4	25.8	55.8	169.4	28.1	66.4	44.9	40.1	63.7	97.0	206.5
**NIR20**	4.4	45.2	35.2	50.2	368.3	62.7	183.2	63.4	19.6	236.3	29.7	20.3	90.8	17.2	43.2	28.9	16.4	44.6	82.1	179.5
**NIR21**	9.0	72.6	38.6	61.0	402.7	64.1	154.4	103.2	47.1	221.2	30.1	25.1	116.8	24.9	67.2	46.6	24.4	46.6	112.6	142.3
**NIR22**	7.2	49.1	31.6	50.3	340.1	49.7	130.9	117.5	30.5	157.6	26.1	19.4	141.9	20.8	60.7	41.5	30.4	58.5	99.2	185.2
**NIR23**	5.7	50.8	38.5	57.3	427.1	72.2	178.7	88.5	30.3	203.1	31.9	20.7	247.2	19.5	54.5	44.5	41.8	83.6	120.7	148.3
**NIR24**	7.5	83.1	33.9	59.2	462.6	65.0	160.3	91.1	50.6	388.6	50.9	25.5	166.5	23.5	51.3	48.7	31.3	68.1	117.7	253.8
**NIR25**	6.9	56.2	38.0	50.3	465.0	64.9	171.1	100.8	38.9	199.0	28.1	30.7	148.9	18.7	51.8	44.7	33.2	65.1	107.2	223.9
**NIR26**	9.6	83.1	46.5	61.1	451.5	69.9	217.9	242.3	33.7	372.9	41.0	33.7	174.2	24.8	56.8	48.0	37.7	61.0	96.9	235.5
**NIR27**	6.7	67.3	39.4	75.6	371.5	58.5	177.1	112.8	55.7	258.0	31.7	20.4	169.3	20.6	48.0	39.9	39.5	70.9	113.6	128.3
**NIR28**	7.0	56.0	47.7	69.1	397.5	69.1	209.8	125.7	35.8	248.0	38.1	27.7	137.0	26.9	55.4	52.6	27.9	59.5	96.7	177.4
**NIR29**	5.6	60.4	39.4	60.6	349.2	63.1	160.5	111.9	37.4	233.6	27.9	27.4	111.0	16.4	45.7	40.5	20.8	41.7	95.9	94.9
**NIR30**	7.0	75.3	32.7	62.6	330.0	60.6	177.5	132.7	39.5	403.1	34.3	40.9	141.8	19.5	50.4	40.8	39.4	60.5	96.5	218.1

**NIR31**	4.1	47.9	30.7	48.8	389.9	58.9	155.4	97.4	28.5	200.1	22.0	29.8	111.9	16.8	49.1	38.0	28.2	43.8	92.9	140.4
**NIR32**	6.5	61.2	38.5	49.4	353.2	57.4	217.6	131.8	31.6	241.8	26.5	21.5	138.2	23.9	43.6	42.2	26.4	46.9	94.5	150.9
**NIR33**	6.0	62.5	46.7	92.4	480.1	67.7	262.2	125.8	43.4	242.2	23.6	21.6	151.9	21.7	48.4	49.7	45.6	70.2	109.5	165.1
**NIR34**	5.3	43.8	47.5	74.8	539.4	74.2	196.0	145.2	45.4	285.6	35.2	34.6	138.6	21.0	47.0	44.2	35.4	69.5	124.1	133.7
**NIR35**	6.8	97.8	41.4	57.4	468.0	73.1	161.6	193.7	50.7	391.8	45.4	23.2	223.8	26.1	68.1	48.4	58.7	87.5	140.2	178.9
**NIR36**	11.3	75.3	40.2	77.0	358.7	67.9	111.4	143.3	48.2	425.6	43.3	32.2	181.9	24.6	55.0	47.1	48.8	77.2	125.2	251.2

Supplementary Table 1. Concentration values of the amino acid measurements from the 47 follicular fluid samples. Abbreviations: ASP: aspartic acid, GLU: glutamic acid, ASN: asparagine, SER: serine, GLN: glutamine, HIS: histidine, GLY: glycine, THR: threonine, ASN: asparagine, ALA: alanine, TYR: tyrosine, CYS: cysteine, VAL: valine, MET: methionine, TRP: tryptophane, PHE: phenylalanine, ILE: isoleucine, LEU: leucine, LYS: lysine, PRO: proline
